# Health literacy and quality of life among people in semi-urban and urban areas

**DOI:** 10.1590/1980-220X-REEUSP-2021-0495

**Published:** 2022-04-13

**Authors:** Celalettin Cevik, İbrahim Kayabek

**Affiliations:** 1 Balikesir University, Faculty of Health Sciences, Department of Public Health Nursing, Balikesir, Turkey.

**Keywords:** Health Literacy, Quality of Life, Suburban Population, Urban Area, Letramento em Saúde, Qualidade de Vida, População Suburbana, Área Urbana, Alfabetización en Salud, Calidad de Vida, Población Suburbana, Área Urbana

## Abstract

**Objective:**

to investigate health literacy level, quality of life and related factors in semi-urban and urban areas.

**Method:**

this cross-sectional study was carried out between December 2018 and February 2019 with 595 participants. The variables found significant in the bivariate regression analysis were included in the multivariate regression analysis.

**Results:**

according to the scores obtained from the Health Literacy Scale, participants’ health literacy 76.5% levels were adequate. The factors affecting the Health Literacy Scale score in semi-urban areas were educational status, income status, presence of a chronic disease, perceived health, and understanding the health information provided. The factors affecting the Health Literacy Scale score in urban areas were age, marital status, reading habits, presence of a chronic disease, and understanding the health information provided (p < .05). There was a statistically significant difference between participants living in semi-urban and urban areas in terms of their health literacy and quality of life levels (p < .001).

**Conclusion:**

the health literacy level was inadequate in three out of ten participants, and it was even lower in semi-urban areas.

## RESUMO

**Objetivo:**

investigar o nível de letramento em saúde, qualidade de vida e fatores relacionados em áreas semiurbanas e urbanas.

**Método:**

este estudo transversal foi realizado entre dezembro de 2018 e fevereiro de 2019 com 595 participantes. As variáveis encontradas significativas na análise de regressão bivariada foram incluídas na análise de regressão multivariada.

**Resultados:**

de acordo com os escores obtidos na Health Literacy Scale, os níveis de letramento em saúde dos participantes de 76,5% estavam adequados. Os fatores que afetaram a pontuação da Health Literacy Scale em áreas semiurbanas foram escolaridade, renda, presença de doença crônica, percepção de saúde e compreender as informações de saúde fornecidas. Os fatores que afetaram a pontuação da Health Literacy Scale em áreas urbanas foram idade, estado civil, hábitos de leitura, presença de doença crônica e compreender as informações de saúde fornecidas (p < 0,05). Houve uma diferença estatisticamente significativa entre os participantes que vivem em áreas semiurbanas e urbanas em termos de letramento em saúde e níveis de qualidade de vida (p < 0,001).

**Conclusão:**

o nível de letramento em saúde foi inadequado em três dos dez participantes, sendo ainda menor nas áreas semiurbanas.

## RESUMEN

**Objetivo:**

investigar el nivel de alfabetización en salud, calidad de vida y factores relacionados en áreas semiurbanas y urbanas.

**Método:**

este estudio transversal se realizó entre diciembre de 2018 y febrero de 2019 con 595 participantes. Las variables que se encontraron significativas en el análisis de regresión bivariante se incluyeron en el análisis de regresión multivariante.

**Resultados:**

según los puntajes obtenidos en la Health Literacy Scale, los niveles de alfabetización en salud de los participantes del 76,5% fueron adecuados. Los factores que afectaron los puntajes de la Health Literacy Scale en áreas semiurbanas fueron la educación, los ingresos, la presencia de enfermedades crónicas, la salud percibida y comprender la información de salud proporcionada. Los factores que afectaron el puntaje de la Health Literacy Scale en áreas urbanas fueron la edad, el estado civil, los hábitos de lectura, la presencia de una enfermedad crónica y comprender la información de salud proporcionada (p < 0,05). Hubo una diferencia estadísticamente significativa entre los participantes que vivían en áreas urbanas y semiurbanas en términos de alfabetización en salud y niveles de calidad de vida (p < 0,001).

**Conclusión:**

el nivel de alfabetización en salud fue inadecuado en tres de cada diez participantes, y fue aún más bajo en las áreas semiurbanas.

## INTRODUCTION

Low health literacy (HL) often has an association with poor health outcomes such as low self-efficacy levels, increased mortality, poor health status and reduced quality of life. HL is the ability to make decisions on health-related issues, and to access, understand and use information sources to protect and improve health and quality of life^(1)^. Low HL can have numerous negative impacts on health, and is associated with decreased adherence to treatment and use of preventive services, an increased number of hospitalizations, health system costs^(2)^, poorer health^(3)^, and higher mortality risk^(4)^. In the literature, the inadequate or problematic HL level ranges between 24.5% and 69.4%^(5,6,7,8,9,10)^. In a study conducted in the USA, participants’ HL 36% levels were below the basic HL level^(11)^. In the literature, the HL level was low in people in the older age group^(10,11)^, employment status^(3,9)^, with low educational status(^3,6,7,9,10^) and with income lower than expenses^(3,6,9,10)^, and in women^(12)^. Quality of life is the perception of one’s own life in a culture and value system according to their own goals, expectations, and standards, and it is a concept used to assess an individual’s or society’s physical and mental state^(13)^. In the literature, the SF-36 Quality of Life Scale Physical Functioning Subscale score ranges between 43.7 and 73.7^(14, 15, 16, 17)^, and the SF-36 Quality of Life Scale Mental Health Subscale score varies between 37.20 and 64.3^(14,15)^. Quality of life scores are low in older adults^(14)^, women^(18)^, people with low education level^(17)^, unmarried people^(19)^, and people with poor income perception^(20)^. One of the predictive factors of both HL and SF-36 is the place of residence within the framework of geographical and economic conditions. For instance, people living in semi-urban areas are disadvantaged in terms of HL and SF-36 because they cannot access health services easily, the number of older adults living there is high, and they have low educational and income status^(8,16)^. A study conducted with 913 women living in a semi-urban region of China showed that decreased HL was associated with decreased quality of life, but only in certain ethnic groups^(21)^.

As for urban areas, the predictive factors of HL and SF-36 are different from those in semi-urban areas: the ability to access correct health information and service, poor ability to make use of health services, and inadequate social skills. Although HL and quality of life are important concepts in healthcare, the link between them is unclear, especially for a population of frequent users of healthcare services in disadvantageous groups. On the other hand, because the number of community-based studies addressing these two concepts, which are extremely important for individuals to lead a healthy life from a perspective of com¬paring two different regions, is limited in Turkey, we decided to perform this study.

In the present study, we aimed to investigate HL levels and quality of life of people living in semi-urban and urban areas, and related factors in Balikesir, a province located in western Turkey.

## METHOD

### Study Design

This cross-sectional study was carried out in two neigh-borhoods located in semi-urban and urban areas of Balikesir province, between December 2018 and February 2019.

### Population

In the study, an urban neighborhood and a semi-urban neighborhood were chosen because we wanted to investigate HL and quality of life levels of people living urban and semi-urban areas, and factors affecting their HL and quality of life levels. Of these two settlements, Bakacak neighborhood has a population of 1,006, who make their living through agriculture and animal husbandry. Bakacak, where traditions affect everyday life, is within the borders of Karesi district in the metropolitan municipality of Balikesir, 15 km from the city center and therefore within easy reach^(22)^. Aygoren neighborhood, the other settlement, where 1,342 people live, is located in urban areas within the borders of Karesi district in the metropolitan municipality of Balikesir. It is one of the first settlements in the city center with historical buildings, and is close to health institutions^(23)^.

### Sample Definition

The study population included 1,906 people aged >15 years. The sample size was calculated in the Epi Info program (prevalence: 27.2%^(7)^, deviation: 4%, design effect: 1.5, confi-dence level: 95%) as 595 people who were contacted using the multi-stage cluster sampling method.

### Data Collection

Data were collected by two researchers using the face-to-face interview technique between September 2018 and February 2019. In sampling, the cluster sampling method proportional to populations in the two districts was used, and 30 clusters each of which included 20 people were reached in accordance with a guideline. In each cluster, the cluster leader household was randomly selected. Moreover, another household was chosen as an alternate cluster leader. Then, a survey was conducted by starting with the cluster whose household was the leader and by skipping the second house and visiting the third house in a row, and one of the households aged >15 at each home visited was interviewed using the systematic sampling method. Then, when the number of households reached 20, the same procedure was conducted in another cluster. If there were not people aged 15 and over at home or if people refused to participate in the study, the survey was continued with the next household. If there were more than one person aged > 15 years at home, of them, the one whose birthday was closest to the day on which the interview was held was interviewed.

Dependent variables were HL and quality of life in health, whereas independent variables were the factors related to sociodemographic characteristics and use of healthcare services. The researchers collected study data through face-to-face interviews by administering the Health Literacy Scale-32 (HLS) and SF-36 Quality of Life Questionnaire (SF-36).

Personal information form items included in the form ques¬tion participants’ sociodemographic characteristics, psychosocial factors, perceived health, use of healthcare services and satisfac¬tion with healthcare services^(11,16,17)^.

HLS was developed based on the European Health Literacy Scale. The items are rated on a 5-point Likert type scale^(7)^. The scale has two healthcare-related subscales (treatment, and disease prevention/health promotion) and four main proces¬ses (accessing the information, understanding the information, assessing the information and using/not using the information). While the scores ranging between 0 and 25 indicate inadequate HL, the scores between >25 and 33 indicate problematic - limited HL. The scores between >33 and 42 indicate adequate HL, and the scores between >42 and 50 indicate excellent HL.

The SF-36 scale used to assess quality of life consists of eight subscales and two summary components (Physical Component Summary and Mental Component Summary). The minimum and maximum possible scores to be obtained from each subscale are 0 (worst health) and 100 (best health), respectively^(22)^.

In the study, participants were asked whether they were able to understand health-related information about HL and quality of life. They were asked to choose the yes option ^(1)^ if they generally understood the information given by health personnel, such as physicians/nurses who provided training/information to them in any health institution, otherwise to choose the no option ^(2)^.

### Data Analysis and Treatment

The SPSS 25.0 package program was used for analysis. Descriptive findings in the study were presented as numbers, percentages and arithmetic mean. In the study, bivariate regres-sion analysis and multivariate regression analysis were used. The variables found significant in the bivariate regression analysis were included in the multivariate regression analysis. P-values considered statistically significant were <0.20 in the bivariate regression analysis and <0.05 in the multivariate regression analysis. Both bivariate and multivariate regression analyses were used to assess the relationship between HLS and quality of life scores with independent variables.

### Ethical Aspects

Before the study was conducted, an ethics committee approval was obtained from the Clinical Research Ethics Committee of the Balikesir University Faculty of Medicine (dated November 07, 2018, numbered 2018/167).

## RESULTS

The distribution of descriptive characteristics of the study group is presented in Table 1.

**Table 1.** Participants’ sociodemographic characteristics – Balikesir, Turkey, 2021.

Sociodemographic characteristics	Semi-urban area n %*	Urban area n %*
**Sex**	
Women	106 (37.1)	170 (55.0)
Men	180 (62.9)	139 (45.0)
**Age**	
15–64 years	265 (92.7)	300 (97.1)
≥65 years	21 (7.3)	9 (2.9)
**Marital status**	
Married	180 (62.9)	158 (51.1)
Single	106 (37.1)	151 (48.9)
**Educational status**	
Primary school or lower	101 (35.3)	51 (16.5)
Junior high school	59 (20.6)	45 (14.6)
Senior high school	86 (30.1)	129 (41.7)
University or higher	40 (14.0)	84 (27.2)
**Occupation**	
Homemaker	68 (23.8)	52 (16.8)
Worker	107 (37.4)	117 (37.9)
Retiree	14 (4.9)	18 (5.8)
Student	42 (14.7)	92 (29.8)
Government official	8 (2.8)	21 (6.8)
Storekeeper	10 (3.5)	7 (2.3)
Farmer	37 (12.9)	2 (0.6)
**Social security**	
No	105 (36.7)	58 (18.8)
Yes	181 (63.3)	251 (81.2)
**Income status**	
Income less than expenses	156 (54.5)	151 (48.9)
Income equal to expenses	109 (38.1)	112 (36.2)
Income more than expenses	21 (7.4)	46 (14.9)
**Reading**	
Never	147 (51.4)	81 (26.2)
Sometimes	90 (31.5)	137 (44.3)
Often	49 (17.1)	91 (29.4)
**Understanding information about health**	
Yes	205 (71.7)	239 (77.3)
No	81 (28.3)	70 (22.7)
Total	286 (100.0)	309 (100.0)

*Column percentage. Note: (N = 595).

Comparison of HLS and SF-36 by region. The mean scores participants obtained from the HLS, and the SF-36 physical and mental components were 31.4 ± 9.3, 70.4 ± 19.5 and 64.2 ± 18.9, respectively. Participants living in semi-urban areas obtained lower scores from HLS and SF-36 scales than did participants living in urban areas (p < 0.05). HLS levels were inadequate in 23.5% of all participants, 29.4% of participants living in semi-urban areas, and 18.1% of participants living in urban areas. While the SF-36 physical component score was higher in participants living in urban areas (p < 0.05), the mental compo¬nent score is similar in participants living in both areas.

The univariate analysis demonstrated that the HLS score was affected by education, income status, presence of a chro¬nic disease, perceived health, and understanding the health information provided in semi-urban areas, and age, marital status, health insurance, reading habits, presence of a chronic disease, and understanding the health information provided in urban areas. On the other hand, of the factors, age, sex, marital status, occupation, health insurance and reading habits in semi-urban areas, and sex, education, occupation, income status and perceived health in urban areas did not affect the HLS score (Table 2).

**Table 2:** Factors affecting the HLS score in semi-urban and urban areas according to bivariate and multivariate regression analysis – Balikesir, Turkey, 2021.

			Semi–urban areas							Urban areas				
Variables	B	Std. Beta	Bivariate analysis		Multivariate analysis			B	Std. Beta	Bivariate analysis		Multivariate analysis		
			95% CI	p	95% CI	p	Adj. R^2^			95% CI	p	95% CI	p	Adj. R^2^
**HLS**														
**Age**	–0.01	–0.01	–0.1; 0.1	.832				–0.17	–0.26	–0.2; –0.1	.003**	–0.2; –0.1	<001	
**Sex**	–1.27	–0.06	–3.8; 1.2	.323				0.31	0.01	–1.5; 2.1	.735			
**Marital status**	1.26	0.06	–1.6; 4.1	.393				–2.84	–0.16	–5.4; –0.2	.032**	–5.1; –0.4	.019	
**Educational status**	1.37	0.14	–0.1; 2.8	.067*	0.4; 2.5	.005		–0.23	–0.02	–1.3; 0.8	.679			
**Occupation**	–0.40	–0.08	–0.9; 0.1	.165*		.126		0.30	0.05	–0.4; 1.0	.420			
**Income**	3.02	0.18	1.3; 4.7	<.001**	1.3; 4.6	<.001		0.30	0.02	–0.9; 1.5	.634			
**Health insurance**	–0.88	–0.04	–3.1; 1.4	.450			0.39	1.63	0.07	–0.6; 3.9	.157*		.084	0.42
**Reading habits**	–0.66	–0.04	–2.5; 1.1	.480				1.31	0.11	0.1; 2.5	.044**	0.1; 2.6	.028	
**Presence of a chronic disease**	4.24	0.17	1.2; 7.2	.006**	1.5; 6.9	.002		–2.56	–0.09	–5.6; 0.5	.107*	–6.0; –0.1	.048	
**Perceived health**	–4.77	–0.30	–6.6; –2.9	<.001**	–6.6; –3.0	<.001		–1.22	–0.07	–3.1; 0.7	.217			
**Understanding the health information provided**	–5.40	–0.23	–7.8; –2.9	<.001**	–7.8, –3.1	<.001		–5.90	–0.29	–8.0; –3.7	<.001**	–8.1; –4.0	<.001	

*p < 0.20, **p < 0.05. Note: (N = 595).

The variables found significant in the bivariate regression analysis were analyzed with the multivariate regression analysis. The factors affecting the HLS score in semi-urban areas were educational status (β = 1.37; 95% CI 0.4, 2.5), income status (β = 3.02; 95% CI 1.3, 4.6), presence of a chronic disease (β = 4.2; 95% CI 1.5, 6.9), perceived health (β = -4.77; 95% CI -6.6, -3.0), and understanding the health information provided (β = 5.4; 95% CI -7.8, -3.1).

The factors affecting the HLS score in urban areas were age (β = -0.17; 95% CI -0.2, -0.1), marital status (β = -2.84; 95% CI -5.1, -0.4), reading habits (β = 1.31; 95% CI 0.1, 2.6), presence of a chronic disease (β = -2.56; 95% CI -6.0, -0.1), and understanding the health information provided (β = -5.90; 95% CI -8.1, -4.0) (Table 2).

The SF-36 physical component score was affected by age, sex, educational status and perceived health in semi-urban areas, and age, sex, health insurance, reading habits, presence of a chronic disease and perceived health in urban areas. However, of the factors, marital status, occupation, income status, health insurance, reading habits, presence of a chronic disease and understanding the health information provided in semi-urban areas, and marital status, educational status, occupation, income status and understanding the health information provided in urban areas did not affect the SF-36 physical component score.

The factors affecting the SF-36 physical component score in semi-urban areas were age (β = -0.47; 95% CI -0.6, -0.4), sex (β = -6.70; 95% CI -11.6, -3.8), and perceived health (β = -10.2; 95% CI -15.2, -7.9). The factors affecting the SF-36 physical component in urban areas were age (β = -0.20; 95% CI -0.4, -0.1), and sex (β = -3.34; 95% CI -7.6; -0.7), health insurance (β = 5.42; 95% CI 1.7; 10.3), reading habits (β = 2.59; 95% CI 0.3, 5.2), and perceived health (β = -9.39; 95% CI -13.5; -5.9) (Table 3).

**Table 3:** Factors affecting the SF-36 physical component score in semi-urban and urban areas according to bivariate and multivariate regression analysis – Balikesir, Turkey, 2021.

		Semi–urban areas						Urban areas				
Variables	B	Bivariate analysis		Multivariate analysis			B	Bivariate analysis		Multivariate analysis		
		95% Cl	p	95% Cl	p	Adj. R^2^		95% Cl	p	95% Cl	p	Adj. R^2^
**Age**	–0.47	–0.6; –0.2	<.001**	–0.6; –0.4	<.001		–0.20	–0.4; 0.1	.071*	–0.4; –0.1	.002	
**Sex**	–6.70	–11.3; –2.0	.005**	–11.6; –3.8	<.001		–3.34	–6.9; 0.2	.070*	–7.6; –0.7	.018	
**Marital status**	–0.83	–6.1; 4.4	.755				–2.39	–7.5; 2.7	.356			
**Educational status**	1.95	–0.7; 4.6	.151*		.166		1.30	–0.8; 3.4	.237			
**Occupation**	0.31	–0.7; 1.3	.554				0.80	–0.6; 2.2	.286			
**Income**	–1.15	–4.2; 1.9	.464				1.16	–1.3; 3.6	.359			
**Health insurance**	–0.65	–4.8; 3.5	.759			0.26	5.42	0.9; 9.8	.017**	1.7; 10.3	.006	0.48
**Reading habits**	–0.75	–4.1; 2.6	.663				2.59	0.1; 5.1	.044**	0.3; 5.2	.025	
**Presence of a chronic disease**	–1.45	–6.9; 4.0	.604				–5.59	–11.7; 0.5	.074*	–11.9; –0.2	.061	
**Perceived health**	–10.2	–13.6; –6.9	<.001**	–15.2; –7.9	<.001		–9.39	–13.2; –5.5	<.001**	–13.5; –5.9	<.001	
**Understanding the health information provided**	–2.46	–6.8; 1.9	.272				1.31	–2.8; 5.4	.532			

*p < 0.20, **p < 0.05. Note: (N = 595).

The variables that affected the score participants obtained from the SF-36 mental component were sex, marital status, health insurance and perceived health in semi-urban areas, and age, sex, educational status and perceived health in urban areas. However, of the factors, marital status, occupation, income status, health insurance, presence of a chronic disease and understanding the health information provided in semi-urban areas, and age, educational status, occupation, income status, reading habits, presence of a chronic disease and understanding the health information provided in urban areas did not affect the SF-36 mental component score.

The factors affecting the SF-36 mental component score were age (β = -0.29; 95% CI -0.4, -0.1), sex (β = -6.29; 95% CI -10.5, -2.0) and perceived health (β = -6.09; 95% CI -9.7, -2.8) in semi-urban areas, and sex (β = -4.50; 95% CI -9.0, -0.7) and perceived health (β = -9.31; 95% CI -14.9, -7.0) in urban areas (Table 4).

**Table 4:** Factors affecting the SF-36 physical component score in semi-urban and urban areas according to bivariate and multivariate regression analysis – Balikesir, Turkey, 2021

			Semi–urban areas							Urban areas				
Variables	B	Std. Beta	Bivariate analysis		Multivariate analysis			B	Std. Beta	Bivariate analysis		Multivariate analysis		
			95% Cl	p	95% Cl	p	Adj. R^2^			95% Cl	p	95% Cl	p	Adj. R^2^
**Age**	–0.29	–0.24	–0.5; –0.1	.006**	–0.4; –0.1	.002		–0.15	–0.10	–0.4; 0.1	.238			
**Sex**	–6.29	–0.15	–11.2; –1.3	.013**	–10.5; –2.0	.004		–4.50	–0.12	–8.7; –.02	.040**	–9.0; –0.7	.020	
**Marital status**	–1.02	–0.02	–6.6; 4.6	.723				–6.26	–0.16	–12.3; –0.2	.043**		.091	
**Educational status**	2.46	0.13	–0.4; 5.3	.092*		.067		–0.22	–0.01	–2.7; 2.3	.865			
**Occupation**	–0.19	–0.02	–1.3; 0.9	.734				0.81	0.06	–0.9; 2.5	.364			
**Income**	–1.21	–0.04	–4.5; 2.1	.473				–1.01	–0.03	–3.9; 1.9	.502			
**Health insurance**	–0.11	–0.00	–4.5; 4.3	.961			0.42	4.41	0.09	–0.8; 9.7	.102*		.138	0.16
**Reading habits**	–3.03	–0.11	–6.6; 0.6	.102*		.061		0.04	0.00	–2.9; 3.0	.977			
**Presence of a chronic disease**	–0.83	–0.01	–6.7; 5.0	.780				–1.22	–0.02	–8.5; 6.0	.740			
**Perceived health**	–6.09	–0.20	–9.6; –2.4	<.001**	–9.7; –2.8	<.001		–9.31	–0.25	–13.8; –4.7	<.001**	–14.9; –7.0	<.001	
**Understanding the health information provided**	–2.67	–0.06	–7.3; 2.0	.266				–1.93	–0.04	–6.8; 2.9	.440			

*p < 0.20, **p < 0.05.

## DISCUSSION

In the present study, in one of the first community-based studies in which HLS and SF-36 were assessed together in semi-urban and urban areas, the HLS score was inadequate in 23.5% of participants and adequate in 36.5% of participants. Our review of studies in the literature demonstrated that the inadequate or highly problematic HLS levels ranged between 24.5% and 69.4%^(5,6,7,9,10)^. The HLS scores in the aforementioned studies were higher than were those in our study, which might be due to the fact that our study was community-based, that the mean age of participants in our study was lower, and that the number of healthy participants in our study was higher^(3)^. In a community-based study conducted in 2016, the HLS score was similar to that in our study^(7)^. In our study, HLS scores demonstrated that the HLS level was inadequate in 29.4% of participants living in semi-urban areas, and in 18.1% of participants living in urban areas. In studies in the literature, the inadequate or problematic HLS levels ranged between 38.1% and 80.6%^(1,23)^ in semi-urban areas and between 44% and 67.6% in urban areas^(23)^. This indicates that the HLS level in these studies was higher in urban areas but lower in semi-urban areas than was that in our study. The results of community-based studies conducted in Iran and China^(21,24)^ were consistent with those of our study. Low HLS level in semi-urban areas than in urban areas may be related to social determinants, culture, age and education^(8)^. It is necessary to carry out studies to improve people’s HL levels, and the primary responsibility falls on nurses, who are members of one of the professions in close contact with people in society. In order for nurses to fulfill their health education and counseling roles effectively, it is necessary to improve both individuals’ and society’s HL levels in the region they serve and reduce regional differences in HL.

In the present study, the mean score participants obtained from the SF-36 physical component was 70.4 ± 19.5. Our search for community-based studies investigating the quality of life of people with a health problem, students or members of a certain profession demonstrated that the number of such studies was limited in the literature^(1,7,24)^. In community-based studies, the mean score for the SF-36 physical component varied between 50.0 ± 9.9 and 75.2 ± 16.4^(13,17,20)^. These results are relatively similar to those of our study. The physical component score in literature was lower than was that in our study, which may have been due to the fact that in the literature, participants were from a socioeconomically disadvantaged area^(20)^. In the present study, the scores obtained from the SF-36 physical component by participants living in semi-urban areas were lower than were those obtained by participants living in urban areas. In a study conducted in Turkey, with a method similar to that in our study, the scores obtained from the SF-36 physical component by participants living in semi-urban areas were lower than were those obtained by participants living in urban areas^(20)^. This might be due to the fact that those living in semi-urban areas were older. In the present study, the mean score participants obtained from the SF-36 mental component was 64.2 ± 18.9. Similarly, in community-based studies in the literature, the mean score for the SF-36 mental component ranged between 50.0 ± 0.9 and 78.8 ± 15.8^(13,17,18,20)^. In our study, the mean scores obtained from the SF-36 mental component by participants living in both areas were similar. Improving quality of life and reducing regional differences are important factors for enhancing society’s health. Therefore, while the planning of health services/care focusing on quality of life can be improved, regional differences can be minimized by nurses’ mediator role.

In our study, according to the results of the multivariate analysis, the factors affecting the HLS scores of participants living in semi-urban areas were educational status, income status, presence of a chronic disease, perceived health, and the understanding the health information provided. In the literature, in studies conducted in semi-urban areas, the factors affecting the HLS scores were age^(10,21,23,24)^, educational status^(1,6,9,10,21,24)^, occupation^(9,21,24)^, income status(6,9,10,21,23,24), and hospitalization status^(24)^. In a study conducted in a semi-urban area, age and sex did not affect the HLS score, which was consistent with our study^(1)^. In our study, the factors affecting the HLS score of participants living in urban areas were age, marital status, rea-ding habits, presence of a chronic disease, and the understanding the health information provided. In the literature, similar to the current study, in studies conducted in urban areas, the factors affecting the HLS scores were age^(5,7,11)^, profession^(24)^, monthly income^(23,24)^, and educational status^(5,7,12,24)^. Different from studies in the literature, other factors affecting the HLS score in our study were the presence of a chronic disease, reading habits, understanding the health information provided, and perceived health. This may be due to the fact that educated people had high levels of awareness of the importance of regu¬lar follow-up for a chronic disease and that they had favorable opinions about the quality of health education they were given. According to the results of our study, the social determinants of health revealed vulnerable groups in terms of HL. Nurses and other healthcare workers should be aware of the disadvantaged groups in terms of HL in society and should make efforts to reduce their disadvantages.

In the present study, the factors affecting the mean score participants obtained from the SF-36 physical component were age, sex and perceived health in semi-urban areas, and age, sex, health insurance, reading habits, and perceived health in urban areas. Similar to our study, in studies in the literature, the factors affecting the scores for the HLS scale physical compo¬nent were age^(14,20)^, sex^(15,20,25)^, and perceived health^(26)^. However, in our study, two other factors which affected the HLS score physical component were health insurance and reading habits. Having health insurance is more important for people living in a semi-urban area, which might be related to the fact that participants living in semi-urban areas were farmers and had voluntary insurance.

In the present study, the factors affecting the mean score participants obtained from the SF-36 mental component were age, sex and perceived health in semi-urban areas, and sex and perceived health in urban areas. In the literature, age^(13,14)^, sex^(15,20,25)^ and perceived health^(26)^ are among the factors affecting the mean score for the SF-36 mental component^(17)^. Quality of life is low in vulnerable groups. In our study, differences between urban and semi-urban areas manifested themselves in mental and physical components. These differences and disadvantaged groups should be taken into account while health services are planned and carried out, and healthcare is provided.

### Strengths and Limitations

Our study has some limitations and strengths. Among the limitations is that the semi-urban region is a village close to the city center, that there is no other village in the periphery, and that the discussion was made horizontally due to the limited number of studies carried out on this issue in the literature. Among the strengths of our study is that it is a community¬based study, that the sample included people from two different areas (semi-urban and urban), that both HLS and SF-36 variables were investigated together and that it is one of the first studies performed within this framework in Turkey.

## CONCLUSION

In the present study, three out of ten participants had inadequate HL. The rate was a third in semi-urban areas and one-fifth in urban areas, while the mean score for the SF-36 physical component was high, and it was above average for the mental component. HLS and SF-36 scores were low in participants living in semi-urban areas. HLS scores were low in older adults, in those with poor health perception, those who were single, those with a chronic disease, those who did not understand the health education provided, those who lacked reading habits, and those with low education and income levels. SF-36 scores were low in older adults, women participants and those with poor health perception. With this issue in mind, it is recommended that programs to improve HLS and SF-36 levels should be carried out by giving priority to disadvantaged groups such as older adults, those with low education, and those with poor economic status. The present study revea¬led the importance of social determinants between regions. Therefore, attempts to eliminate differences between regions should be made, and disadvantaged groups should be enabled to access qualified healthcare. Furthermore, plans should be made to increase SF-36 levels of healthy individuals besides their HLS levels.

Nurses, especially public health nurses, play a critical role in protecting and improving individuals’, families’ and society’s health. It is important to assess individuals’ HL and quality of life so that nurses can effectively fulfill their independent roles in providing health education and counseling. It is a fact that the result of this assessment will contribute to the adoption of practices related to the protection and development of health through the training and counseling to be provided in accordance with an individual’s HL level. Due to factors such as individuals’ health status, inequality in accessing health oppor-tunities and health information and increases in costs, planning and implementation of initiatives to improve people’s HL levels gain importance. Revealing the status and determinants of quality of life in health and HL will reduce inequality in accessing health opportunities and improve health.

## ASSOCIATE EDITOR

Cristina Lavareda Baixinho
